# How Public Health Nurses Identify and Intervene in Child Maltreatment Based on the National Clinical Guideline

**DOI:** 10.1155/2014/425460

**Published:** 2014-11-19

**Authors:** Paavilainen Eija, Helminen Mika, Flinck Aune, Lehtomäki Leila

**Affiliations:** ^1^School of Health Sciences (Nursing Science), University of Tampere, Etelä-Pohjanmaa Hospital District, Finland; ^2^School of Health Sciences, University of Tampere and Science Center, Pirkanmaa Hospital District, Finland; ^3^School of Health Sciences, University of Tampere, National Institute of Health and Welfare, Technologies and Practices Assessment Unit, FinSoc, Finland; ^4^The Finnish Union of Public Health Nurses, Finland

## Abstract

*Objectives*. To describe how Finnish public health nurses identify and intervene in child maltreatment and how they implement the National Clinical Guideline in their work. *Design and Sample*. Cross-sectional survey of 367 public health nurses in Finland. *Measures*. A web-based questionnaire developed based on the content areas of the guideline: identifying, intervening, and implementing. *Results*. The respondents reported they identify child maltreatment moderately (mean 3.38), intervene in it better (4.15), and implement the guideline moderately (3.43, scale between 1 and 6). Those with experience of working with maltreated children reported they identify them better (*P* < 0.001), intervene better (*P* < 0.001), and implement the guideline better (*P* < 0.001) than those with no experience. This difference was also found for those who were aware of the guideline, had read it, and participated in training on child maltreatment, as compared to those who were not aware of the guideline, had not read it, or had not participated in such training. *Conclusions*. The public health nurses worked quite well with children who had experienced maltreatment and families. However, the results point out several developmental targets for increasing training on child maltreatment, for devising recommendations for child maltreatment, and for applying these recommendations systematically in practice.

## 1. Background

Child maltreatment is a public health problem and a violation of children's human rights [1]. The latest research in Finland [2, 3] shows that children and youth experience a wide range of maltreatment at home-meaning physical, emotional, and sexual violence, neglect, and witnessing violence between parents. The same forms of child maltreatment are observed in other countries in Europe and globally [4–8]. In addition, research attention has been paid to children living in families where intimate partner violence is part of their everyday life [2, 9]. Although much has been done for discovering the situation of children living in violent homes, effort is still needed for knowing more about identification and prevention practices, for developing them.

Researchers in the child maltreatment field show that child maltreatment within the family has an enormous effect on children and their future physical, emotional, and social welfare, often resulting in inequality and marginalization [10, 11]. A meta-analysis on the health consequences [12] stressed that all forms of child maltreatment should be considered important risks to health. Lifelong impairments in learning, behaviour, and both physical and mental health are strongly linked to adverse experiences in childhood. Exposure to child maltreatment can disrupt normal biological and social development, creating a cascade of events that lead to toxic stress which results in changes in the developing nervous, cardiovascular, immune, and metabolic systems which last a lifetime [13]. Awareness of the serious long-term consequences should encourage better identification of those at risk and the development of effective interventions to protect children from violence [1, 14]. In the UK, the National Institute for Health and Clinical Excellence (NICE) guidance was developed to raise healthcare professionals' awareness of the alerting features of child maltreatment [15]. In Finland a guideline has also been written [10] concerning identifying and intervening in child maltreatment, based on a systematic literature review [16]. In the Finnish guideline, the risk factors for the child, the parents, and the family, signs and symptoms, and the principles for identifying them and intervening in child maltreatment are described. The central means of identifying and also intervening in maltreatment include, for example, knowing and evaluating the signs of maltreatment of a child, discussion with their parents about the family situation and relationships within the family, and discussion about child rearing practices, home visits, and multiprofessional practices [10]. The guideline is meant to be a tool for evidence-based practice [17, 18], used by public health nurses (PHNs) and in multiprofessional collaboration with other professionals meeting and working with children, adolescents, and families in different settings. In papers evaluating multiprofessional practices, the knowledge exchange concerning the situation of the child and the family is frequently ineffective or not family oriented: professionals may not actively include the family in the collaboration or they do not work intensively enough together (e.g., see [19, 20]). Identifying and intervening in families' high-risk situations and child maltreatment is not as evidence-based as it could be.

PHNs, as healthcare workers who meet almost all children and their families at clinics, schools, and homes, are key persons in identifying, preventing, and intervening in child maltreatment. In Finland PHNs provide care for children and families across a wide age range, from maternity care and preschool to school-aged children, and in many different settings including clinics, clients' homes, and schools. Before school age (age 7 in Finland), children and their families visit child health clinics at least 16 times and, once in school, children and young people will see a PHN at least once a year. These services are free for all families and almost all families use them [21].

The objective of the current research was to describe how Finnish PHNs identify and intervene in child maltreatment and how they implement the clinical guideline concerning these issues in their work. Findings can be used for the development of identification and intervention practices and education.

## 2. Research Questions

This study was designed to address the following research questions.

1. How do PHNs identify child maltreatment?
2. How do they intervene in child maltreatment?
3. How do they describe their implementation possibilities concerning identifying and intervening in child maltreatment?
4. What are the background factors that promote identification, intervention, and implementation?

## 3. Methods

### 3.1. Design and Sample

The respondents to the current survey were recruited from the register of the Finnish Union of Public Health Nurses, in 2012. All the members who had an email address and appeared to have worked in child-related clinics (*n* = 800) were sent the electronic questionnaire in February 2012 and were reminded after two weeks. The board of the union gave the ethical approval and research permission. Completing the survey indicated informed consent and no identifiers were collected [22]. In the letter attached to the questionnaire, it was stated that the permission for the research was clear, the results will be published anonymously and filling in the questionnaire was interpreted as participation in the study. Altogether 367 PHNs answered the survey, resulting in the response rate of 46%.

### 3.2. Measures

The survey instrument was developed for this study, and its content was based on the National Clinical Guideline concerning identifying and intervening in child maltreatment and implementing the guidelines on this topic [10, 16]. It included five background questions (gender, age, work experience, present job and location, and work experience in cities/countryside/both) and three questions concerning whether they knew that guidelines existed and, if so, whether they had read them and whether they had been trained on the topic. They were also asked how many child maltreatment cases they had encountered or suspected, during the previous six months. The survey instrument consisted in Likert Scale statements (6 = totally agree, 1 = totally disagree) divided under three sum variables:* identifying* (8 statements),* intervening* (31 statements), and* implementing* (8 statements). The instrument was developed by the group of researchers (*n* = 12) who were experts in child maltreatment issues and/or statistical methods. The instrument was pilot tested by sending the electronic version to ten PHNs who completed the questionnaire. Modifications were made based on their responses.

### 3.3. Data Analysis

The means and standard deviations (SDs) or percentage distributions were calculated for all demographic variables. Responses for nurses' perceptions of how they identify and intervene in child maltreatment and how they implement the guidelines were divided into groups: disagree (responses of 1–3) and agree (responses 4–6). These three variables were also combined as sum variables,* identifying* (Cronbach's Alpha 0.866),* intervening* (0.957), and* implementing*  (0.854), showing solid reliability [23]. Differences between groups according to demographics were tested using Pearson correlations, *t*-tests, and ANOVA. Linear regression analysis was also done to see which factors explain* identifying, intervening, *and* implementing*. Having worked at maternity and family planning, child health clinic, school health, and all other places were used as separate independent binary variables in the model, together with suspicion of maltreatment (or actual meeting of maltreated children) and working years.

## 4. Results

### 4.1. Demographic Characteristics

Almost all of the respondents (*N* = 367) were women (there were only two men), with a mean age of 42 (ranging from 23 to 64) and a mean working career of 12 years (ranging from 0 to 37 years). Thirty eight percent of them worked in family planning and maternity, 45% in a child welfare clinic, 30% in school health, and 48% in other clinics, for instance in student health care clinics or clinics for adults. The respondents may work in several clinics; for instance 107 respondents worked in both maternity and child welfare clinics. Seventy-seven percent knew that there was a guideline, 46% reported they had read it, and 46% had participated in training on the topic. During the previous six months, 37% of the respondents reported they had not met maltreated children, 20% had met 1–4 maltreated children, and 5% had met five or more maltreated children, to the best of their knowledge. Correspondingly, 28% reported they had not suspected any maltreatment cases, 36% had suspected that 1–4 children they encountered had been maltreated, and 7% had suspected that five or more children had been maltreated (Table 1).

### 4.2. Identifying Child Maltreatment

The PHNs agreed they were able to identify maltreated children moderately well (mean 3.38, SD 0.84). Only 43% agreed the child's behaviour was a factor in identifying child maltreatment, and 37% agreed that the parents' behaviour was a factor. Fifty-four percent agreed that physical signs were a factor and 44% agreed that psychological signs were a factor. Only 15% of the PHNs meet maltreated children often, according to their own evaluation (Table 2).

### 4.3. Intervening in Child Maltreatment

Intervening in child maltreatment was easier for the respondents (mean 4.15, SD 0.91) than identifying it (mean 3.38). Most (80%) respondents felt they discussed everyday problems and problems in the child's development adequately (80%) and that they advised the parents to seek help when they need it (88%). On the other hand, the respondents reported that couple's relationship problems are discussed less often (52%). The respondents thought they helped the maltreated child (52%) and the family (50%) sufficiently well. Fifty-nine percent thought that multiprofessional collaboration was working well in their municipality, and 50% of them receive enough support for multiprofessional collaboration from their superiors. Fifty-four percent of the respondents have joint guidelines for child maltreatment cases, and 65% have clear instructions on how to make a report to child protection authorities (Table 3).

### 4.4. Implementing the Clinical Guideline

PHNs reported they implemented the recommendations written in the guideline moderately (mean 3.43, SD 1.01). Among the respondents, 87% considered the guidelines important. The guideline for child maltreatment guides the work of 57% of the respondents, and 87% reported they will gladly adjust their work practices according to the guideline. Of the respondents, 21% had received enough training regarding the guideline and 44% had studied the content of the guideline independently. Twenty-three percent of the respondents had discussed the guideline at their workplace, 44% supported each other in actions following the guideline, and 38% thought that their workplace had sufficient resources for acting according to the guideline.

### 4.5. Factors Promoting Identifying, Intervening, and Implementing

PHNs who had met maltreated children reported they were able to identify child maltreatment better than those without that experience, according to their own evaluation (mean 3.79 versus 3.07, *P* < 0.001). They also intervened in child maltreatment cases better (mean 4.48 versus 3.88, *P* < 0.001) and implemented the recommendations of the guideline better (mean 3.79 versus 3.16, *P* < 0.001). The same applied to nurses who were aware of the guideline, had read it, and participated in training on recognizing and intervening in child maltreatment (Table 4).

According to the regression analysis, with working years and suspicion of maltreatment as additional independent variables, those working as school health nurses were able to identify cases better than others. In addition, those working as school health nurses or at child welfare clinics also intervened better than others. Those working at family planning or maternity clinics implemented the guideline better than others. Those who had suspected child maltreatment obviously had better identification, intervention, and implementation than those who had not suspected child maltreatment (Table 5).

According to the regression analysis, with working years and actual contact with maltreated children as additional independent variables, only intervention was at a higher level (for those who worked as school health nurses) compared to others. Those who had met maltreated children obviously had better identification, intervention, and implementation than those who had not met maltreated children.

## 5. Discussion

The PHNs identified child maltreatment to a moderate degree. They thought that they identified risk factors related to the child, parents, or the family the best and issues related to the child's or the parents' behavior the worst. They identified signs of physical and emotional abuse better. Similar results have also been reported in a survey for hospital staff [24]. Different forms of child maltreatment are identified at different levels: signs of physical maltreatment are often clearer than, for example, signs of emotional maltreatment or neglect.

The respondents thought that they can intervene in maltreatment better than they can identify it. They discussed both everyday issues and problems related to child development with the parents at a child health center, but they failed to discuss relationship issues sufficiently. These results also agree with earlier results [9, 25–27].

When suspecting that a child has been maltreated, the respondents asked about it directly, made a child protection report, and documented the event in the child's documentation. In their opinion, however, they felt that they were not able to provide enough help for the child and the family in the situation. According to our regression models, those nurses working at schools assessed their child maltreatment identification practices more positively than those working at other places. This may be because children's behavior or problems in school attendance may more likely lead to discussions about the child's situation than at a child welfare clinic. At clinic children are younger and do not express being ill so clearly. At family planning or maternity clinics nurses implemented the guideline very well at a knowledge level and were willing to use this knowledge. However, at the identifying and intervening levels, they were not able to apply their willingness in real situations. According to earlier studies [9, 25, 27], PHNs feel that they are in a good position to take the actions needed but they need more training on applying their knowledge in real situations when working with children and families.

According to the PHNs, multiprofessional collaboration is not working very well in the municipality or organization where they work. Common guidelines on how to act on child maltreatment suspicions or how to make a child protection report were not used often enough. Some of the results may seem to be confusing, for example, concerning agreeing on the importance of the guidelines even when not knowing them very well. This may be due to the fact that the respondents agree that identification and intervening are important but they do not know well enough what to do and how to implement their knowledge into multiprofessional work. According to Lehtomäki [17], the attitudes towards care guidelines in general are positive but applying them requires support and administrative effort from their superiors and development of joint practices. In this study, the respondents rated themselves as not receiving enough support from their superiors or child protection authorities. According also to Yagasaki and Komatsu [28], guidelines are regarded as important; however they are not fully applied into practice. To be successful in applying any research-based guidelines, organizational, administrational, multidisciplinary, and individual barriers have to be challenged by a strategy that gives tools for effective implementation.

PHNs who had met maltreated children or who had participated in training on child maltreatment were able to identify maltreatment and they intervened in it better than those who had not met such children or participated in such training. Earlier studies have also gained similar results. According to Paavilainen et al. [24], nurses who have participated in training or who have met maltreated children find identification and intervention even more difficult than nurses who have not encountered these issues at all. Based on this, it is possible that nurses who have explored the issue and acted on it have a more profound understanding of how difficult and complex the issue is. Also, when asking about these difficult issues with a cross-sectional design as we did in our study, using a self-reported questionnaire might also be a limitation. However, it seems that PHNs did not over estimate their capability in identifying or intervening and many developmental challenges can be presented based on the results.

### 5.1. Implications for Nursing Practice

PHNs are generally aware that identification of and intervention in child maltreatment are an important part of their job (see also [25]). However, the lack of effort in creating common guidelines and practices and ensuring the functionality of multiprofessional collaboration were weaknesses in practical work and actions [19]. This is a question of work organization and management and also of focusing on activities that are really helpful and effective for children and families. PHNs are in a position to take a leadership role in the prevention of child maltreatment as well as addressing the system barriers such as knowledge exchange challenges or coordination within multiprofessional activities. This will lead to supporting families before risks of maltreatment are realized into actual maltreatment in families. Solving or treating child maltreatment cases is the responsibility of many administratively separate units: child health centers, hospitals, at day care, and in child protection. The managers of these units should ensure that collaboration, in both prevention and treatment levels, can work across administrative boundaries. This kind of development can be done globally, based on international research evidence and on the service system of children and families in each country.

### 5.2. Implications for Nursing Education

More attention should be paid to training on child maltreatment in the basic and complementary education of PHNs. This training should include the topics of high risk family environments (identifying and discussing them), discussing the everyday issues and problems of families, the risks and manifestations of maltreatment, the identifying symptoms and signs, and the concrete means and methods to identify and intervene in maltreatment. There should also be training on issues regarding multiprofessional collaboration and legislation. The service system and legislation concerning children and families differ in each country; this has of course to be considered. However, the health, well-being, and needs of children are global issues and can be used as the basis of education.

### 5.3. Implications for Nursing Research

These results show well the situation concerning the identification of and intervention in child maltreatment by PHNs and how they implement the guideline concerning these issues in their work with children and families. Further knowledge is needed, for example, on how to provide education on these issues and change PHNs' practices. This could be done by a follow-up study with an educational intervention. Also, comparable data from other countries would be interesting and important, providing knowledge of the situation in different countries. This study is already in process, and there is already data from Japan collected with the same instrument.

## 6. Conclusion

The primary prevention of child maltreatment is the most important issue from the perspectives of children, families, and the whole society, in Finland and globally, although also secondary and tertiary prevention are very crucial to development. In aiming to prevent child maltreatment and increase the well-being of children, guidelines and risk assessment practices for PHNs and supportive interventions for families with child maltreatment risk should be developed and evaluated. This can be done by using guidelines for creating effective interventions and evaluating the process and outcomes by follow up research.

## Figures and Tables

**Table 1:** Background information concerning the participants (*N* = 367).

Variables	Mean (SD)
Age in years	42,5 (10,8)
Working years as a nurse	12,3 (9,9)

	Percentages

Working in urban areas/countryside/both	65,9/26,7/7,4
Knowledge of the existence of a guideline (yes/no)?	77,1/22,9
Had they read the guideline (yes/no)?	46,3/53,7
Had they had education on the topic (within a year/earlier/never)?	11,2/34,6/54,2
How many (0/1–4/5 or more//missing) child maltreatment cases they had met during six months?	36,8/19,9/4,6//38,7
How many (0/1–4/5 or more//missing) suspected child maltreatment cases they had met during six months?	28,3/35,7/6,5//29,4

**Table 2:** Items included in *identifying* sum variable and the percentages that agree/disagree (*N* = 367).

Identifying	(agree 4–6/disagree 1–3)
PHNs meet maltreated children often	15/85%
PHNs recognize child maltreatment based on	
Child-related risk factors	53/47%
Risk factors related to parents	66/34%
Family-related risk factors	67/33%
The child's behavior	43/57%
The parents' behavior	37/63%
PHNs recognize physical signs sufficiently well	54/46%
PHN's recognize mental signs sufficiently well	44/56%

**Table 3:** Items included in *intervening* sum variable and the percentages that agree/disagree (*N* = 367).

Intervening	(agree 4–6/disagree 1–3)
PHNs discuss sufficiently well with families about	
Risk factors in families	61/39%
Child rearing practices	69/31%
Problems in the couple's relationship	52/48%
Problems in everyday life	80/20%
Child development	80/20%
Problems in child development	79/21%
PHNs advice parents sufficiently well to	
Seek help when needed	88/12%
Act well in situations when the child has a tantrum	76/24%
Act well when the child behaves badly	71/29%
Act well when the child does not fulfill expectations	60/40%
Act well when the child has special needs or is ill	69/31%
Act well when the child cries	76/24%
Discuss their joint child rearing practices	67/33%
When suspecting child maltreatment, PHN	
Asks about it straightforwardly	69/31%
Always makes a child welfare notification	86/14%
Helps the maltreated child sufficiently well	52/48%
Helps the family sufficiently well	50/50%
Documents maltreatment sufficiently well	82/18%
Guides to follow-up treatment sufficiently well	81/19%
Listens to the family under suspicion sufficiently well	83/17%
Collaborates sufficiently well with other professionals	86/14%
Thinks multiprofessional collaboration works well in the municipality	59/41%
Thinks multiprofessional collaboration works well in their organization	69/31%
Knows who to contact when suspecting child maltreatment	87/13%
When suspecting child maltreatment PHN gets enough support from	
Superiors	50/50%
Peers	82/18%
The clinic physician	69/31%
Child protection	69/31%
In our clinic:	
We have joint instructions to handle child maltreatment cases	54/46%
We have clear instructions on how to make a child welfare notification	65/35%
It is possible to work according to the child maltreatment guideline	59/41%

**Table 4:** The effects of separate background factors on* identifying, intervening*, and *implementing*.

Variables	Identifying	Intervening	Implementing
Age (correlation, significance)	0,01 NS	0,01 NS	0,14^**^
Working years as a nurse (correlation, significance)	−0,02 NS	−0,03 NS	0,07 NS
Working in urban areas/countryside/both (ANOVA sig.)	NS	NS	NS
Knowledge of the existence of a guideline (*t*-test sig.)	∗∗∗	∗∗∗	∗∗∗
PHN had read the guideline (*t*-test sig.)	∗∗∗	∗∗∗	∗∗∗
PHN had had education on the topic (ANOVA sig.)	∗	∗∗∗	∗∗∗
How many (0, 1−4, 5, or more) child maltreatment cases they had met during six months (ANOVA sig.)	∗∗∗	∗∗∗	∗∗∗
How many (0, 1–4, 5 or more) suspected child maltreatment cases they had met during six months (ANOVA sig.)	∗∗∗	∗∗∗	∗∗∗

Estimate significance (*P* value): NS = not significant;  ^*^<0,05;  ^**^<0,01;  ^***^<0,001.

**Table 5:** Estimated unstandardized regression coefficients for all variables from linear regression models, separately for *identifying, intervening*, and* implementing* (*N* = 367).

Model variables	Identifying	Intervening	Implementing
Have worked in: maternity and family planning	0,075	0,127	0,296
Have worked at: a Child Health Clinic	−0,103	0,325	0,309
Have worked at: a School Health Clinic	0,216	0,301^*^	0,133
Have worked at: other places	0,086	−0,381^*^	0,044
Working years	0,000	−0,003	0,012
Had met maltreatment cases (yes/no)	0,658^***^	0,390^**^	0,583^***^

Have worked in: maternity and family planning	−0,058	0,227	0,344^*^
Have worked at: a Child Health Clinic	−0,008	0,309^*^	0,096
Have worked at: a School Health Clinic	0,293^*^	0,316^**^	0,068
Have worked at: other places	−0,038	−0,317^**^	−0,106
Working years	−0,005	0,000	0,015^*^
Had suspected maltreatment cases (yes/no)	0,507^***^	0,322^**^	0,310^*^

Estimate significance (*P* value):  ^*^<0,05;  ^**^< 0,01;  ^***^<0,001.
